# Extended Thymectomy via Thoracoscopy and Cervical Incision in a Child with Anterior Mediastinal Mixed Germ Cell Tumor and Paraneoplastic Precocious Puberty

**DOI:** 10.70352/scrj.cr.25-0596

**Published:** 2025-12-09

**Authors:** Masaya Yamoto, Yu Sugai, Yousuke Gohda, Hiromu Miyake, Akiyoshi Nomura, Koji Fukumoto, Shinichiro Sano, Hideto Iwafuchi, Kenichiro Watanabe

**Affiliations:** 1 Department of Pediatric Surgery, Osaka City General Hospital, Osaka, Osaka, Japan; 2 Department of Surgery, Shizuoka Children’s Hospital, Shizuoka, Shizuoka, Japan; 3 Department of Endocrinology and Metabolism, Shizuoka Children’s Hospital, Shizuoka, Shizuoka, Japan; 4 Department of Pathology, Shizuoka Children’s Hospital, Shizuoka, Shizuoka, Japan; 5 Department of Hematology and Oncology, Shizuoka Children’s Hospital, Shizuoka, Shizuoka, Japan

**Keywords:** anterior mediastinal tumor, mixed germ cell tumor, precocious puberty, thoracoscopic extended thymectomy, pediatric surgery

## Abstract

**INTRODUCTION:**

Anterior mediastinal germ cell tumors are uncommon in children, and mixed germ cell tumors containing malignant components are particularly rare. These tumors may occasionally present with endocrine manifestations such as gonadotropin-independent precocious puberty caused by ectopic human chorionic gonadotropin (hCG) secretion. Complete resection is the mainstay of treatment, and extended thymectomy is recommended when the tumor involves or is inseparable from the thymus. Minimally invasive techniques such as video-assisted thoracoscopic surgery (VATS) have been increasingly applied, and in some adult cases, a cervical incision has been combined to ensure removal of the cranial thymic extension. However, pediatric reports of VATS combined with a cervical incision for extended thymectomy are lacking.

**CASE PRESENTATION:**

We report a 9-year-old boy who presented with gonadotropin-independent precocious puberty characterized by suppressed gonadotropins, elevated serum testosterone, and increased hCG and alpha-fetoprotein levels. Imaging demonstrated a 45 × 25-mm anterior mediastinal mass with features suggestive of teratoma and suspicious cranial solid components. The tumor displaced the thymus cranially into the lower cervical region, prompting the decision for extended thymectomy via a combined thoracoscopic and cervical approach. A small transverse cervical incision was used to mobilize the cranial thymus, and thoracoscopic dissection allowed safe control of thymic veins and arteries and complete en bloc resection of the thymus and tumor. The specimen measured 6 × 5 × 3 cm and weighed 53 g. Histopathology revealed a mixed germ cell tumor composed of mature teratoma and seminoma. The postoperative course was uneventful, tumor markers normalized, and the patient subsequently received adjuvant cisplatin-based chemotherapy. He remains disease-free at follow-up.

**CONCLUSIONS:**

To our knowledge, this case represents the 1st pediatric anterior mediastinal mixed germ cell tumor treated with thoracoscopic extended thymectomy combined with a cervical incision. The approach allowed safe and complete resection despite cranial displacement of the thymus, while avoiding sternotomy. Recognition of endocrine manifestations such as hCG-induced precocious puberty is essential for early diagnosis. This case demonstrates that minimally invasive extended thymectomy with cervical extension is feasible in children and may be applied to achieve radical resection of complex anterior mediastinal tumors.

## Abbreviations

AFP
: alpha-fetoprotein
DHEA-S
: dehydroepiandrosterone sulfate
FSH
: follicle-stimulating hormone
hCG
: human chorionic gonadotropin
LH
: luteinizing hormone
SIOP
: International Society of Paediatric Oncology
VATS
: video-assisted thoracoscopic surgery

## INTRODUCTION

Primary anterior mediastinal tumors in children are uncommon, and germ cell tumors account for a significant subset. Among them, teratomas are most frequent, whereas mixed germ cell tumors containing malignant components such as seminoma or choriocarcinoma are rare.^1–4)^ Clinical manifestations may include endocrine abnormalities such as precocious puberty or gynecomastia due to ectopic secretion of hCG.^3,5,6)^ Recognition of such endocrine features is crucial, as they may serve as early diagnostic clues. Complete surgical excision remains the cornerstone of treatment for anterior mediastinal germ cell tumors.^1)^ Extended thymectomy, involving en bloc removal of the thymus with perithymic fat, was historically established for myasthenia gravis but is also applicable in oncologic contexts. Minimally invasive approaches such as VATS have gained popularity in adult practice, demonstrating safety and efficacy comparable to sternotomy in selected cases.^7–10)^ Furthermore, several groups have described combined transcervical and thoracoscopic or mediastinoscopic approaches to achieve complete extended thymectomy, including removal of cervical extensions of thymic tissue.^8,11)^ However, in pediatric populations, such combined approaches are rarely reported, and most published cases describe standard thoracoscopic resections for teratomas.^12,13)^. Herein, we report a child with an anterior mediastinal mixed germ cell tumor presenting with hCG-induced precocious puberty, who underwent successful thoracoscopic extended thymectomy combined with a cervical incision.

## CASE PRESENTATION

**Fig. 1:**
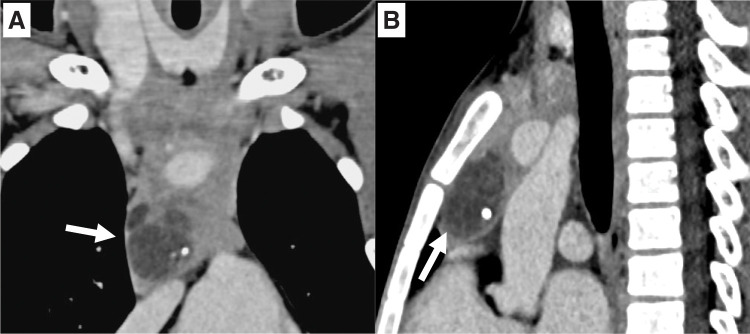
Contrast-enhanced chest CT. (**A**) Coronal view showing an anterior mediastinal mass with cystic components (white arrow). (**B**) Sagittal view demonstrating the cystic portion of the tumor (white arrow).

**Fig. 2:**
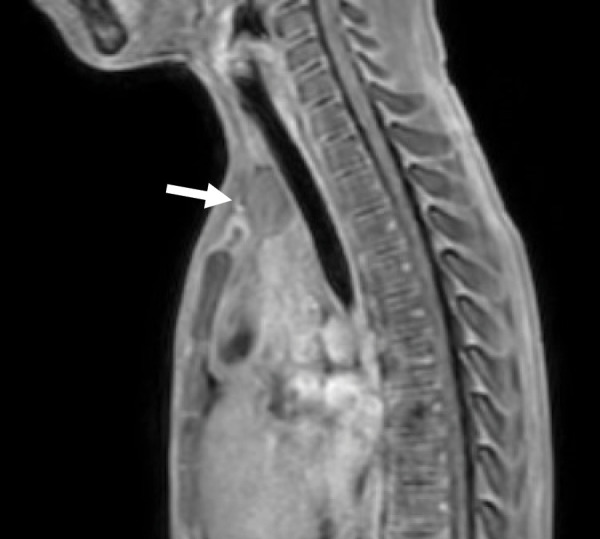
MRI. MRI showing cranial displacement of the thymus into the lower neck (white arrow). A solid cranial component adjacent to the teratomatous portion appears suspicious for malignant transformation.

A 9-year-old boy was referred to Shizuoka Children’s Hospital with suspected precocious puberty, presenting with voice changes, pubic hair development, and rapid growth. Laboratory evaluation revealed suppressed gonadotropins (LH <0.30 mIU/mL, FSH <0.30 mIU/mL), paradoxically elevated serum testosterone (2.05 ng/mL), and increased levels of hCG (7.6 mIU/mL) and AFP (13.3 ng/mL), findings consistent with gonadotropin-independent precocious puberty. Serum somatomedin-C (354 ng/mL) and DHEA-S (30 μg/dL) were within normal ranges for age. Imaging studies demonstrated a 45 × 25-mm anterior mediastinal mass located beneath the thymus, composed predominantly of cystic elements with calcification and fat, findings suggestive of a teratoma (**Fig. 1A** and **1B**). In the cranial portion, however, irregular solid components were present in the central thymus, raising—but not confirming—the suspicion of malignant transformation. The tumor displaced the normal thymic tissue cranially, causing the upper pole of the thymus to extend into the lower neck (**Fig. 2**). Based on these findings, extended thymectomy was planned to achieve both diagnostic and therapeutic complete resection, considering that definitive malignancy could not be established preoperatively.

**Fig. 3:**
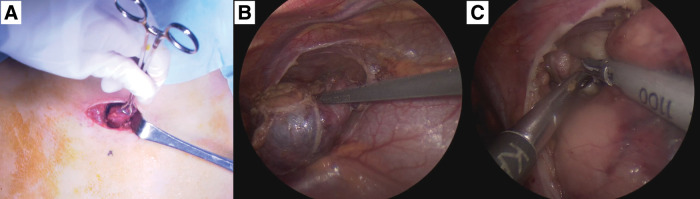
Intraoperative findings. (**A**) Dissection of the cranial portion of the tumor through a cervical incision. (**B**) Circumferential incision of the mediastinal pleura anterior to the phrenic nerve, followed by mobilization of the thymus. (**C**) Tumor dissection was completed by connecting the thoracoscopic and cervical approaches.

The patient underwent thoracoscopic tumor resection combined with a cervical mini-incision under general and epidural anesthesia. The operation began with a 4-cm transverse cervical incision above the sternum (**Fig. 3A**). The cranially displaced thymic apex, pushed upward by the tumor, was carefully mobilized and dissected from surrounding cervical tissues while preserving the sternocleidomastoid muscle and protecting the trachea and major vessels. After securing the cervical portion, thoracoscopic ports were inserted at the 4th intercostal mid-axillary line, the 5th intercostal anterior and mid-axillary lines, and the 6th intercostal anterior chest wall. The mediastinal pleura was incised anterior to the phrenic nerve, and dissection proceeded circumferentially around the thymus and tumor (**Fig. 3B**). Caudal dissection was performed along the pericardium, while cranial dissection was carried out along the innominate vein. During this process, several thymic veins draining into the innominate vein were identified, carefully clipped, and divided. A large tributary from the junction of the innominate and right internal jugular veins was controlled with clips before transection. Four additional thymic veins were also divided, and both right and left thymic arteries were controlled—on the right using harmonic scalpel and on the left by clip ligation at their origin from the internal thoracic artery. These steps allowed progressive mobilization of the tumor from surrounding vital structures. The tumor was noted to be adherent to adjacent thymic lobules, and its cranial extension into the lower neck confirmed the necessity of combining the cervical incision with thoracoscopic dissection (**Fig. 3C**). After complete mobilization, attempts to deliver the mass through the cervical wound were unsuccessful due to its size and friability. Consequently, the thoracoscopic port site at the 5th intercostal space was extended, and the specimen was retrieved using an endoscopic retrieval bag. Mediastinal and right pleural drains were placed via the 4th and 6th intercostal port sites. Hemostasis was secured, and all wounds were closed in multiple layers. The entire thymus with tumor was removed en bloc, achieving complete macroscopic resection without injury to major vessels or phrenic nerves. Intraoperative blood loss was minimal, and the patient tolerated the procedure well (**Supplementary Video**).

**Fig. 4:**
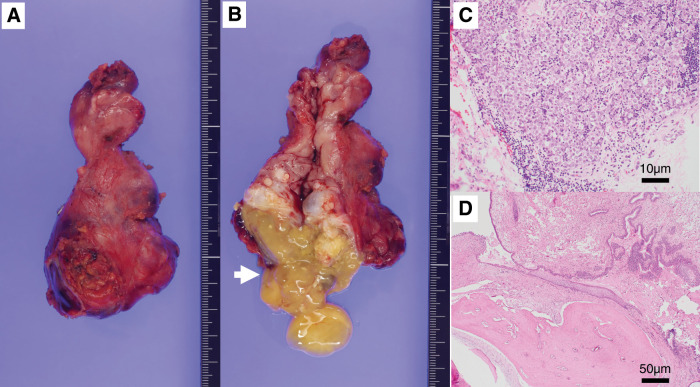
Resected specimen and histopathological findings. (**A**) Gross specimen following en bloc extended thymectomy. (**B**) Specimen sectioned along the midline, revealing cystic teratomatous components containing yellowish keratinous material (white arrow), with a cranial solid region corresponding to the germ cell tumor. (**C**) Seminomatous component composed of large atypical cells with clear cytoplasm and prominent nucleoli (hematoxylin and eosin stain). (**D**) Histopathological examination showing mature teratomatous elements, including squamous epithelium, cartilage, and glandular tissue (hematoxylin and eosin stain).

Gross examination of the resected specimen measuring 6 × 5 × 3 cm and weighing 53 g revealed cystic cavities containing keratinous debris and hair (**Fig. 4A** and **4B**). Histopathology confirmed mature teratomatous components, including squamous epithelium, hair follicles, sebaceous glands, cartilage, bone, and glandular epithelium. In addition, atypical cells with clear cytoplasm and prominent nucleoli were observed along the thymic lobules and within fibrous stroma. Immunohistochemical analysis demonstrated positivity for OCT3/4, SALL4, CD117, D2-40, and PLAP, consistent with seminoma, whereas stains for hCG, AFP, glypican-3, and CD30 were negative. The final diagnosis was a mixed germ cell tumor of the anterior mediastinum, composed of teratoma and seminoma, arising from the thymus.

The patient’s postoperative course was uneventful. Tumor markers normalized promptly, and he was discharged in good condition. Considering the seminomatous component, adjuvant chemotherapy with cisplatin, etoposide, and bleomycin was initiated. Treatment was generally well tolerated, with only moderate hematological toxicities, and follow-up imaging demonstrated no evidence of recurrence.

## DISCUSSION

This case highlights several distinctive aspects in the presentation, pathology, and surgical management of pediatric anterior mediastinal germ cell tumors. First, the endocrine presentation was notable. Our patient developed gonadotropin-independent precocious puberty, with elevated testosterone despite suppressed gonadotropins, attributable to ectopic secretion of hCG. Similar endocrine manifestations, including rapidly progressive puberty and gynecomastia, have been reported in association with mediastinal germ cell tumors.^3,5,6)^ Such findings underscore the importance of considering extragonadal germ cell tumors in children presenting with atypical pubertal development.

Second, imaging suggested a complex lesion: a cystic mass with calcification and fat was located beneath the thymus, findings highly suggestive of a teratoma. In contrast, the cranial portion within the central thymus contained irregular solid components that raised—but did not confirm—the suspicion of malignant transformation. The tumor displaced the normal thymic tissue cranially, causing the upper pole of the thymus to extend into the lower neck. Because definitive malignancy could not be established preoperatively, we considered complete resection to be the most appropriate diagnostic and therapeutic approach. In retrospect, if malignancy had been clearly established, preoperative biopsy followed by neoadjuvant chemotherapy might have been an alternative to achieve tumor shrinkage. However, given the imaging characteristics and the feasibility of safe complete resection, a thoracoscopic extended thymectomy combined with a cervical incision was selected. Pathological examination confirmed a mixed germ cell tumor composed of mature teratoma and seminoma arising from thymic tissue. Although mature teratomas are the most frequent mediastinal germ cell tumors in children, seminomatous or mixed variants are rare.^1,2,4)^ The coexistence of teratoma with seminoma has direct implications for management, as malignant components necessitate adjuvant chemotherapy. In our case, cisplatin-based chemotherapy was administered postoperatively, leading to normalization of tumor markers, and the patient remains disease-free on follow-up.

Third, the surgical strategy was carefully adapted to the unique anatomy. The tumor displaced the thymus cranially into the lower cervical region, making a purely thoracoscopic approach insufficient. We therefore adopted a combined thoracoscopic and cervical approach to achieve an extended thymectomy. In this case, complete extended thymectomy was chosen rather than limited tumor excision because the tumor was inseparable from the thymus and extended cranially into the lower cervical region. Extended thymectomy enabled en bloc removal of the tumor and thymic tissue, minimizing the risk of microscopic residual disease or capsule rupture. This approach follows the oncologic principle of radical resection for mediastinal germ cell tumors and represents an adaptation of the extended thymectomy concept, originally established in adult myasthenia gravis surgery, to ensure complete removal of thymic and perithymic tissue in pediatric malignant lesions. Moreover, the decision to perform an extended thymectomy was influenced by the potential for microscopic invasion of thymic parenchyma and perithymic fat. Because the tumor was contiguous with the thymus, limited resection might have left residual thymic tissue or perithymic nests that could harbor microscopic disease. To achieve oncologic radicality, the entire thymus and surrounding fatty tissue were removed en bloc. This strategy parallels the principle of extended thymectomy in adult myasthenia gravis, where complete clearance of thymic and perithymic tissue reduces the risk of residual disease and recurrence. To contextualize our case, we reviewed PubMed/MEDLINE and Google Scholar (1990–2025) for pediatric mediastinal germ cell tumors treated surgically (**Table 1**). Reported cases predominantly involved mature teratomas resected via open or thoracoscopic approaches without a cervical extension. No pediatric case employing a combined thoracoscopic plus cervical extended thymectomy was identified, underscoring the novelty of our surgical strategy.^14–16)^ The concept of extended thymectomy, established in adult myasthenia gravis, has been shown to achieve more complete clearance when a transcervical extension is included.^7–9)^ Novellino et al. 1st described an “extended” thymectomy by cervicotomy and thoracoscopy without sternotomy,^7)^ and subsequent prospective and retrospective studies confirmed the feasibility and immunologic significance of including cervical fat.^8,9)^ Yu et al. further developed combined transcervical and unilateral-thoracoscopic or mediastinoscopic thymectomy, achieving satisfactory outcomes.^10,11)^ Long-term institutional experience has demonstrated that VATS thymectomy can yield results comparable to sternotomy while reducing morbidity.^17)^ While adult series have reported combined transcervical and thoracoscopic (or mediastinoscopic) extended thymectomy, no pediatric cases were identified in our literature search. Pediatric reports have described thoracoscopic or, more recently, robotic-assisted thymectomy, but these did not include a cervical extension.^12,13)^ Our case therefore represents a novel adaptation of an adult surgical principle to a pediatric malignant germ cell tumor with cranial extension. Primary mediastinal malignant teratomas are known to carry a poor prognosis among extragonadal germ cell tumors, particularly when incomplete resection or intraoperative rupture occurs. Therefore, achieving complete en bloc removal without capsule violation is essential for local control and long-term survival. In our case, meticulous dissection was performed to mobilize the tumor together with the thymus, and the specimen was retrieved using an endoscopic bag without rupture or spillage. The combined thoracoscopic and cervical approach allowed safe control of the thymic vessels and ensured an intact, complete resection, which is of particular importance in malignant germ cell tumors.

**Table 1:** Summary of reported pediatric mediastinal germ cell tumors treated by minimally invasive endoscopic surgery

Author (year)	Age/sex	Pathology	Surgical approach	Cervical extension	Outcome/notes
Kojima et al., 2024^14)^	9 y/F	Mature teratoma	Assisted VATS with mini-thoracotomy	No	Complete resection despite SVC adhesion; disease-free at 1 year.
Huang et al., 2025^15)^	2 m/N.D.	Mature cystic teratoma	Complete thoracoscopic resection	No	Infant case; successful minimally invasive excision without complications.
Present case	9 y/M	Mixed GCT (teratoma + seminoma)	Combined thoracoscopic + cervical extended thymectomy	Yes	Complete en bloc resection without rupture; adjuvant chemotherapy; disease-free on follow-up.

F, female; GCT, germ cell tumor; M, male; N.D., not described; SVC, superior vena cava; VATS, video-assisted thoracoscopic surgery

In addition to its surgical and oncologic aspects, the endocrine presentation was a distinctive feature of this case. Elevated serum β-hCG led to secondary activation of the hypothalamic–pituitary–gonadal axis, resulting in precocious puberty. This phenomenon has been described in several pediatric cases of hCG-secreting mediastinal germ cell tumors, in which testosterone elevation and premature virilization typically precede the detection of a mediastinal mass.^3–5)^ In our patient, puberty onset occurred approximately 2 months before tumor diagnosis, and both β-hCG and testosterone levels normalized within 4 weeks after complete tumor removal, consistent with prior pediatric reports.^3–5)^ These findings highlight the reversibility of hCG-induced endocrine activation and underscore the importance of hormonal monitoring for early diagnosis and postoperative follow-up. Postoperative endocrine follow-up showed rapid normalization of serum testosterone and gonadotropins. β-hCG levels became undetectable by postoperative week 3, and both testosterone and LH/FSH returned to prepubertal ranges by week 4. These values remained stable during 1 year of follow-up, indicating durable resolution of paraneoplastic endocrine activity after complete tumor removal. During postoperative surveillance, the patient has remained disease-free for 18 months. Serum AFP and β-hCG levels were measured every 2–3 months and have consistently remained within the normal range since the completion of chemotherapy. No radiologic or biochemical evidence of recurrence has been detected to date, supporting durable remission. Although the tumor was completely resected and met the criteria for Stage I according to the Practical Guidelines for Pediatric Cancer (2016),^18)^ adjuvant chemotherapy with cisplatin, etoposide, and bleomycin (PEB) was administered. This decision was made after multidisciplinary discussion, as the tumor extended cranially into the cervical region and included a seminomatous component, raising concern for potential microscopic spread beyond the thymic capsule. The regimen was selected according to the Japanese Pediatric Germ Cell Tumor Group protocol, which is consistent with international standards such as the AGCT1531 trial by the Children’s Oncology Group and SIOP. PEB remains the standard 1st-line regimen for malignant or high-risk extragonadal germ cell tumors, given its proven efficacy in preventing recurrence through the eradication of occult residual disease. In this case, 4 cycles of PEB were administered without major complications, resulting in sustained normalization of AFP and β-hCG during 18 months of follow-up and durable disease-free survival.

Finally, this case emphasizes the importance of tailoring surgical approaches to tumor location and anatomic distortion in children, where the operative field is small and vital structures are in close proximity. Minimally invasive approaches can be safely applied when combined with adjuncts such as a cervical incision. In the future, robotic-assisted thymectomy may further enhance visualization and precision in confined pediatric mediastinal spaces, though pediatric data remain scarce and no reports of robotic plus cervical approaches are currently available.^13)^

## CONCLUSIONS

We report a rare pediatric case of anterior mediastinal mixed germ cell tumor presenting with gonadotropin-independent, hCG-induced precocious puberty, successfully treated with extended thymectomy via a combined thoracoscopic and cervical approach. This case illustrates that minimally invasive extended thymectomy, when adapted from adult surgical concepts, can achieve complete and radical resection in children with complex anterior mediastinal tumors while minimizing surgical trauma. It also emphasizes the importance of recognizing endocrine manifestations of mediastinal germ cell tumors and tailoring operative strategies to the unique anatomic challenges of pediatric patients.

## SUPPLEMENTARY MATERIALS

Supplementary Video Intraoperative video of extended thymectomy via thoracoscopy and cervical incision.

## DECLARATIONS

### Availability of data and materials

The data that support the findings of this study are available on request from the corresponding author. The data are not publicly available due to privacy or ethical restrictions

## References

[1] Göbel U, Calaminus G, Schneider DT, et al. Management of germ cell tumors in children: approaches to cure. Oncol Res Treat 2002; 25: 14–22. 10.1159/000055197 11893878

[2] König R, Schönberger W, Grimm W. Mediastinal teratocarcinoma and pituitary stalk germinoma in a patient with Klinefelter’s syndrome (in German). Klin Padiatr 1990; 202: 53–6. 1690312 10.1055/s-2007-1025486

[3] Lin CM, Lee CT, Tung YC, et al. Endocrine dysfunction in Taiwanese children with human chorionic gonadotropin-secreting germ cell tumors. J Formos Med Assoc 2014; 113: 102–5. 24530243 10.1016/j.jfma.2012.04.007

[4] Yuri T, Shimano N, Ohashi Y, et al. An autopsy case of primary mixed choriocarcinoma and mature teratoma located in the thymic region associated with elevated human chorionic gonadotropin levels and characteristic testicular changes. Med Mol Morphol 2006; 39: 49–53. 16575515 10.1007/s00795-006-0305-z

[5] Kim SJ, Ko AR, Jung MK, et al. Male patients presenting with rapidly progressive puberty associated with malignant tumors. Ann Pediatr Endocrinol Metab 2016; 21: 51–5. 27104181 10.6065/apem.2016.21.1.51 PMC4835563

[6] Nagi DK, Jones WG, Belchetz PE. Gynaecomastia caused by a primary mediastinal seminoma. Clin Endocrinol (Oxf) 1994; 40: 545–8. 7514514 10.1111/j.1365-2265.1994.tb02496.x

[7] Novellino L, Longoni M, Spinelli L, et al. “Extended” thymectomy, without sternotomy, performed by cervicotomy and thoracoscopic technique in the treatment of myasthenia gravis. Int Surg 1994; 79: 378–81. 7713713

[8] Shigemura N, Shiono H, Inoue M, et al. Inclusion of the transcervical approach in video-assisted thoracoscopic extended thymectomy (VATET) for myasthenia gravis: a prospective trial. Surg Endosc 2006; 20: 1614–8. 16794781 10.1007/s00464-005-0614-7

[9] Vizeteu R, Damian M, Smeu B, et al. Video-assisted thoracoscopic extended thymectomy (VATET) with cervical approach for myasthenia gravis--initial experience (in Romanian). Chirurgia (Bucur) 2010; 105: 797–803. 21355177

[10] Yu L, Ma S, Jing Y, et al. Combined unilateral-thoracoscopic and mediastinoscopic thymectomy. Ann Thorac Surg 2010; 90: 2068–70. 21095376 10.1016/j.athoracsur.2010.02.042

[11] Xue L, Pang X, Zhang Y, et al. Extended thymectomy by a cervical incision additional to bilateral VATS approach. J Vis Surg 2017; 3: 83. 29078646 10.21037/jovs.2017.05.04 PMC5637920

[12] Carter M, Ungerleider S, Goldstein SD. Thymectomy for juvenile myasthenia gravis: a narrative review. Mediastinum 2024; 8: 35. 38881806 10.21037/med-23-41 PMC11176985

[13] Hanke R, Emr B, Taylor M, et al. Robotic resection of a giant thymolipoma in a pediatric patient. J Surg Case Rep 2024; 2024: rjae691. 39512494 10.1093/jscr/rjae691 PMC11541546

[14] Kojima M, Touge R, Kurihara S, et al. Successful mediastinal teratoma resection in a child by assisted VATS: a case report. J Cardiothorac Surg 2024; 19: 511. 39227874 10.1186/s13019-024-03022-0 PMC11370218

[15] Huang H, Chang PCY, Yeh YT, et al. Thoracoscopic resection of a mediastinal mature cystic teratoma in a two-month-old infant: a case report and literature review. Pediatric Health Med Ther 2025; 16: 209–16. 40791251 10.2147/PHMT.S535366 PMC12338318

[16] Keiya T, Uehara H, Aoyagi M, et al. A case of anterior mediastinal mature teratoma with severe inflammatory extension into the neck. Surg Case Rep 2024; 10: 254. 39496873 10.1186/s40792-024-01946-2 PMC11534930

[17] Yablonsky P, Pischik V, Tovbina MG, et al. The results of video-assisted thoracoscopic thymectomies in Saint Petersburg, Russia: 20-year of experience. J Vis Surg 2017; 3: 113. 29078673 10.21037/jovs.2017.06.13 PMC5639043

[18] Japanese Society of Pediatric Hematology/Oncology. Practical guidelines for pediatric cancer (in Japanese). Tokyo: Kanehara; 2016.

